# Paediatricians’ Practice About SUDDEN Infant Death Syndrome in Catalonia, Spain

**DOI:** 10.1007/s10995-016-2225-4

**Published:** 2017-02-03

**Authors:** Federico de Luca, Esperanza L. Gómez-Durán, Josep Arimany-Manso

**Affiliations:** 1 Department of Social Statistics and Demography, University of Southampton, Southampton, SO17 1BJ UK; 2 Professional Liability Service, Col·legi Oficial de Metges de Barcelona, Barcelona, Spain; 3 Department of Medicine, Universitat Internacional de Catalunya, Barcelona, Spain; 4 Forensic and Legal Medicine Unit, Department of Public Health, Universitat de Barcelona, Barcelona, Spain; 5 Psychiatric Unit, Hestia Alliance, Barcelona, Spain

**Keywords:** SIDS, Knowledge management, Paediatricians, Recommendations

## Abstract

*Background* SIDS is the major cause of death among healthy born infants in developed countries. Its causes are still unclear, but its risk can be reduced by implementing some simple active interventions. In Spain, limited attention was given to SIDS by the national healthcare system, and actual data on healthcare professionals’ practice on this topic was not available. This study explored for the first time paediatricians’ knowledge and practice about SIDS. *Methods* A cross-sectional survey was carried out between November 2012 and April 2013 in Catalonia, and reached 1202 paediatricians. The response rate was 46%. *Results* 94% of respondents perceived themselves as qualified for giving advice and recommendations about SIDS to parents, but only 58% recognized the supine position as the safest position and recommended the supine position exclusively to parents. Seniority and ‘having received a specific training about SIDS’ were detrimental to paediatricians’ knowledge. *Discussion* Efforts should be made in order to improve paediatricians’ knowledge and practice about SIDS. Specific refresher trainings are highly recommended, and should especially target paediatricians with higher seniority. These trainings could be provided as optional modules, as we could see that the paediatricians who would most benefit from them are already aware of the need to refresh their knowledge.

## Significance

*What is already known on this subject?* SIDS is the major cause of death among healthy born infants in developed countries. Its causes are still unclear, but it is possible to implement some active interventions in order to reduce its risk. In Spain, limited attention was given to SIDS and actual data on healthcare professionals’ practice on this topic did not exist.

*What this study adds?* This study explores for the first time paediatricians’ knowledge and practice on this topic. 94% of respondents perceived themselves as qualified for giving advice and recommendations about SIDS to parents, but many of them still believe that the side position can be deemed as an acceptable position to be recommended to parents.

## Introduction

The term Sudden Infant Death Syndrome (SIDS) refers to the death of an infant under one year of age which occurs during sleep and whose cause remains unexplained despite a thorough investigation of the case which includes a complete autopsy and a clinical history review (Krous et al. 2004). Although reliable figures are not available for Spain (Grupo de Trabajo de Muerte Súbita Infantil—AEP 2013), SIDS is the major cause of death among healthy born infants in developed countries, with rates that vary between 0.06 and 0.87 per 1000 healthy newborns (Centraal Bureau voor de Statistiek 2015; Mathews et al. 2015). Around 90% of SIDS deaths happen in the first 6 months of life, and boys that are more likely to die than girls (at a ratio of 3:2) (Moon et al. 2007a). Some minorities present statistically significant differences in their rate of SIDS due to different exposures to some SIDS risk factors (e.g. prevalence of supine positioning) (Mathews et al. 2015; Ball et al. 2012). SIDS pathogenesis is due to the convergence of three factors: the critical period of development in which it occurs, the intrinsic vulnerability of the infant and external factors such as the sleep position (Filiano and Kinney 1994). Nevertheless, its exact cause is still unclear (Mitchell 2009), and due to this uncertainty it is still not possible to completely eliminate the risk of SIDS. As a result, great attention has been given to epidemiological findings about it, so that it is now possible to reduce this risk by implementing some simple active interventions. The American Academy of Pediatrics (AAP) considers as ‘*A-level recommendations*’ for reducing the risk of SIDS those presented in Table 1 (Task Force on SIDS 2011a). These recommendations have also been endorsed by the Spanish Paediatrics Society (AEP) in 2013 (Grupo de Trabajo de Muerte Súbita Infantil—AEP 2013).

**Table 1:** AAP ‘*A-level recommendations*’ for the prevention of SIDS (released in 2011)

1. To put the infant to sleep supine on a firm surface and in an environment free of soft objects and loose bedding
2. To avoid overheating of the infant’s room
3. To give infants a pacifier before putting them to sleep
4. To share the same room with the infants but not the bed
5. To breastfeed
6. To receive proper prenatal care for pregnant women
7. To avoid smoking, alcohol and drugs consumption during and after pregnancy
8. To avoid the use of home cardiorespiratory monitors as a strategy for reducing the risk of SIDS
9. To actively involve paediatricians, family physicians and other primary care professionals in the campaigns focused on preventing SIDS

Recently, epidemiologists have started using the broader term Sudden Unexpected Death in Infancy (SUDI) which includes SIDS and other sleep-related deaths (Mitchell and Krous 2015). This shift was mostly due to the fact that some pathologists used the cause of death ‘Unascertained’ rather than ‘SIDS’ (Mitchell and Krous 2015; Huber 1993) as they did not believe SIDS to be a disease entity. In addition, as SIDS has no specific identifiable cause and is a definition of exclusion, child death investigations are limited by the country’s child death review investigation, process and classification systems, and might result in classifying many of these deaths as SUDI but not as SIDS. On the other hand, Krous made a plea to retain use of the term SIDS as it captures the complex interaction of factors that must occur simultaneously to cause death (Krous 2013). Considering Krous’s invitation and the fact that in Spain the debate still focuses on SMSL (which corresponds to the Spanish translation of SIDS), we decided to maintain the term SIDS for the purpose of this paper.

In the last 25 years, many prevention campaigns targeted caregivers and healthcare professionals in the USA in order to increase their knowledge on this topic, and they had a very positive effect (Hauck and Tanabe 2009; Moon et al. 2008, 2004; Moon and Oden 2003). The AAP recommends that ‘*all physicians, nurses, and other health care professionals should receive education on safe infant sleep*’, and suggests that they should develop initiatives that promote adherence to prevention guidelines among their patients (Task Force on SIDS 2011b). In Spain, the first study about SIDS and its risk factors dates back to 1986. In that year, five paediatric hospitals combined together their efforts to select, under common criteria, those infants that were at risk of SIDS, and to enrol them in a program of cardio-respiratory home monitoring (Mesa Redonda ‘*Síndrome de muerte súbita del lactante*’ 1987). Before this one, only a limited number of studies about SIDS had been carried out in Spain, and paediatricians themselves had little knowledge about this topic (Camarasa Piquer 2003). Limited attention was given to SIDS by the national healthcare system and even the national mortality rate attributable to SIDS was not reliable (Camarasa Piquer 1991). In 1991 the Spanish Association of Paediatricians established the Working Group for the Study and Prevention of SIDS, where all the different medical specialties involved in SIDS prevention interacted with each other. All the 12 regional societies of Paediatrics were represented in this Working Group, and they all endorsed the protocols approved by it (Camarasa Piquer 2003). The action of the Working Group and of various prevention campaigns, such as ‘*Ponle a dormir boca arriba*’ (Put them to sleep face up, which was launched in 2000), contributed to a marked improvement in the awareness of SIDS among healthcare professionals and the general public (Camarasa Piquer 2003). However, data about the effect of these campaigns on the rate of SIDS is not available (Grupo de Trabajo de Muerte Súbita Infantil—AEP 2013).

According to the Strategic Plan for Paediatric Primary Care of the Catalonia Health Department, paediatricians have a crucial role in transmitting the SIDS risk reduction message to parents (Generalitat de Catalunya 2007). As a consequence, their knowledge must be as correct and aligned with the latest scientific evidence to the greatest extent as possible, and it is important to be able to evaluate it and to let policy makers know whether it is necessary to improve it. Moreover, healthcare professionals have the responsibility to use this knowledge to guide practice, as it has been proved that what they recommend to parents has a great influence in the subsequent parents’ behaviour at home (Raydo and Reu-Donlon 2005). For this reason, it is important to assess also the quality of the recommendations that they give to parents about the sleep position. However, even if now available for several countries (de Luca and Hinde 2016), actual data on this topic still does not exist for Spain. The aim of this study is to explore for the first time paediatricians’ knowledge and practice about SIDS in a Spanish region. Moreover, we would like to provide local policy makers with useful indications about the strength and weaknesses of paediatricians’ current approach to this subject. To do so, we explore the dissemination of knowledge about SIDS and its risk factors among paediatricians, as well as the recommendations that they give to parents on this topic. This project is the result of a joint effort of the University of Southampton and the Official Colleges of Physicians of Catalonia, and was approved by the Ethics Committee and the Research Governance Office of the University of Southampton (Project ID: 1197). The Official Colleges of Physicians already granted from its members the permission to run similar projects.

## Methods

A cross-sectional survey was carried out between November 2012 and April 2013 in Catalonia. All the provinces of the region (4) were invited to participate in the survey, but only three of them accepted (Barcelona, Tarragona and Lérida). The sample frame was retrieved through the databases of the respective Official Colleges of Physicians, and included all physicians with a registered specialty in Paediatrics. As paediatricians over 70 are no longer allowed to work in the public healthcare system in Catalonia (and are not likely to work in the private sector either), we excluded from the study those aged 71 or more. The survey followed a mixed-mode approach: a first mailing by post containing an invitation letter and a copy of the questionnaire (together with a pre-addressed and pre-stamped return envelope) was followed by three reminders. The first reminder consisted of a thank-you/reminder postcard. The second reminder consisted of an envelope containing a different invitation letter and another copy of the questionnaire (together with another pre-addressed and pre-stamped return envelope). The third reminder consisted of an email containing a final invitation letter and an electronic version of the questionnaire which replicated the one on paper. Two weeks passed between each of the mailings, and no tokens of appreciation were used to increase the response rate. In order to take into proper consideration the linguistic diversity of the region, all letters were sent both in Catalan and in Spanish. As for the questionnaire, the one included in the first mailing was in Catalan, while the one included in the last one was in Spanish. The web questionnaire could be filled in in Catalan or in Spanish according to the respondents’ preferences. All questions were designed with a multiple choice format, and all response options were mutually exclusive. Respondents could give only one answer to each question, except for those about the workplace, the safest sleep position and the recommended sleep position (for which multiple answers were accepted) and those about seniority and the latest training about SIDS (for which they had to fill in a blank).

The questionnaire included questions about the respondents’ rating of their own knowledge and confidence in discussing with parents about this topic, their clinical practice about SIDS, the sleep position which they recommended and 15 questions about the effect of several behaviours on the risk of SIDS. Some of these behaviours did not have an effect on the risk of SIDS (e.g., ‘*Encouraging tummy time when the infant is awake and observed*’) but were included in order to test whether paediatricians critically evaluated each of the items without assuming that they all represented proven risk factors. Information on the demographic and professional background of the respondents was also collected. The questionnaire was based on a previously validated one (de Luca and Boccuzzo 2014) which was updated with additional details such as information about SIDS training (if any), their offspring, the rating of their confidence and knowledge about this topic, and 7 behaviours out of the 15 to be evaluated. At a later stage, though, two of the 15 items were excluded from the data analysis, as it emerged that in the Catalan translation their wording created some confusion among respondents (one item was about the firmness of the mattress and the other was about the temperature of the infant’s room). The percentage of correct answers given to the remaining 13 items was later used as a measure of respondents’ overall knowledge on this topic. Following prevailing ethical principles, participants received written information about the study and response to the survey was considered as consent to participate.

We performed a descriptive analysis of the responses, and correlations (ρ) were tested in order to investigate the relationship between variables. Pearson’s correlation was performed when both variables were continuous; Spearman’s correlation was performed when one variable was ordinal and the other was either continuous or dichotomous; point biserial correlation was performed when one variable was continuous and the other was dichotomous; tetrachoric correlation was performed when both variables were dichotomous. All the statistical analysis was performed in STATA (StataCorp 2011).

## Results

The population of interest consisted of 1,202 paediatricians, distributed between the provinces of Barcelona (996), Tarragona (124) and Lérida (82). The overall response rate was 45.9% (43.2% in Barcelona, 54.0% in Tarragona and 67.1% in Lérida), with a total of 552 responses. 63.2% of the respondents were females and 97.1% obtained their specialty in paediatrics in Spain (Table 2). On average, paediatricians had 24.6 years of professional experience, and in the vast majority of cases (84%) had children of their own. Only 34.4% of paediatricians reported having received specific training about SIDS, and, on average, this training took place 8 years before this survey (with a minimum of 1 year and maximum of 33 years, data not shown in table). The majority of them (54.4%) rated their most recent training about SIDS as satisfactory, while only 2.3% felt unsatisfied with it. 63.8% of the respondents stated that they had a direct experience of a case of SIDS.

**Table 2:** Demographic and professional background of the sample (N = 552)

Variable	Category	% (if not otherwise stated)^a^
Gender	Male	36.8
	Female	63.2
Country of medical specialisation	Spain	97.1
	Other	2.7
	Did not reply	0.2
Seniority	Average (standard deviation)	24.6 (9.72)
	Did not reply	4.7
Workplace	Primary Care Center (CAP)	60.7
*(it was possible to give more than one response) *	Private clinic	15.0
	Private practice	30.3
	Public hospital	27.7
	Private hospital	10.5
Children of their own	None	14.9
	Less than 3 years old	9.2
	More than 3 years old	75.2
	Did not reply	0.7
Received a training about SIDS	Yes	34.4
	No	60.7
	Did not reply	4.9
Rating of most recent training about SIDS	Satisfactory	54.4
	Neither satisfactory nor unsatisfactory	30.0
	Unsatisfactory	2.3
	Did not reply	13.4
Had a direct experience of a case of SIDS	Yes	63.8
	No	35.7
	Did not reply	0.5

^a^Percentages may not add up to 100% because of rounding

Overall, 93.7% of respondents perceived themselves as qualified for giving advice and recommendations about SIDS to parents (data not shown in table). 63.9% of paediatricians rated their knowledge about SIDS and its risk factors as very high or somewhat high, and 1.3% as somewhat low or very low (Table 3). At the same time, 62.3% of respondents rated their confidence in discussing issues related to SIDS with parents as very high or somewhat high, and 2.3% as somewhat low or very low. Almost one in two of paediatricians reported that they informed parents about SIDS ‘*about once a week*’ or more often (49.3%), and 78.5% stated that they talked with parents about the correct sleep position with the same frequency.

**Table 3:** Respondents’ rating of their own knowledge about SIDS and confidence in discussing it with parents, and frequency with which respondents discussed these issues with parents

Variable	Category	%
Rating of their own knowledge about SIDS and its risk factors	Very high	7.4
	Somewhat high	56.5
	Average	34.2
	Somewhat low	1.1
	Very low	0.2
	Did not reply	0.5
Rating of their confidence in discussing issues related to SIDS with parents	Very high	9.6
	Somewhat high	52.7
	Average	35.0
	Somewhat low	1.6
	Very low	0.7
	Did not reply	0.4
Frequency with which respondents discussed SIDS with parents^a^	More than once a week	29.7
	About once a week	19.6
	Less frequently	44.0
	Never	6.3
	Did not reply	0.4
Frequency with which respondents discussed the correct sleep position with parents^a^	More than once a week	55.3
	About once a week	23.2
	Two or three times a month	10.9
	About once a month	4.0
	Less than once a month	5.3
	Never	1.1
	Did not reply	0.4

^a^The possible answers to the questions about the frequency with which paediatricians discussed these issues with parents was different due to space constraints in the questionnaire. After discussion with policy makers, it was agreed that further granularity would have been more meaningful in the question about the sleep position

57.6% of paediatricians recognised the supine position alone as the safest position against SIDS, and 58.1% exclusively recommended the supine sleep position (Table 4). A significant amount of respondents, 35.7 and 37.0% respectively, appeared to consider and recommend side sleeping as appropriate for infants. A minority of respondents, 5.3 and 3.7% respectively, considered and recommended the prone position.

**Table 4:** Respondents’ answers about the safest sleep position and the recommendations given to parents (percentages, respondents could choose multiple positions)

Variable	Category	%^a^
Position that respondents believed to be the safest (N = 552)	Supine exclusively	57.6
	Lateral + lateral and supine	35.7
	Other positions	5.3
	Did not know	0.4
	Did not reply	1.1
Position that respondents recommended to parents (N = 546)	Supine exclusively	58.1
	Lateral + lateral and supine	37.0
	Other positions	3.7
	Did not recommend a specific position	0.2
	Did not reply	1.1

^a^Percentages may not add up to 100% because of rounding

**Fig. 1:**
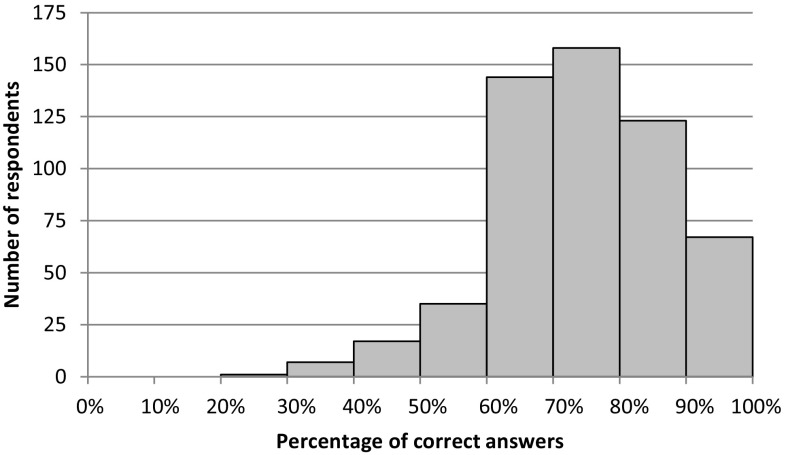
Distribution of respondents in terms of percentage of correct answers given to the 13 items about SIDS risk factors

The evaluation that paediatricians gave about the 13 items describing potential SIDS risk factors is presented in Table 5. A majority of paediatricians correctly evaluated the effect of each item on the risk of SIDS, except for the item about room sharing. In this case, only 31.0% of the respondents recognized that this behaviour lowered the risk of SIDS, while the majority (56.2%) believed that it did not have any effect on it. In the other cases, the percentages of correct answers went from around 90% for the effect of smoking and of prone and supine positions, to less than 50% for the use of pacifiers and room sharing. On average, each paediatrician answered correctly to 75.0% of the items, but only 2.5% of respondents answered all questions correctly. Figure 1 shows how respondents were distributed in terms of percentage of correct answers.

**Table 5:** Respondents’ answers to the effect of different behaviours on the risk of SIDS (percentages, correct answers are given in italics)

	It lowers the risk	It does not affect the risk	It increases the risk	I do not know	Does not reply
Placing infants for sleep in a supine position	*89.0*	2.7	6.9	0.4	1.1
Offering infants a pacifier at nap time and bedtime	*47.8*	31.9	7.1	12.0	1.3
Allowing infants to sleep in the same bed as their parents	3.6	16.5	*73.2*	4.5	2.2
Encouraging tummy time when the infant is awake and observed	17.8	*69.8*	8.0	3.8	0.7
Making up the bedding so that the infant’s feet reach the foot of the crib	12.3	*48.9*	4.5	32.8	1.5
Maternal smoking during pregnancy	0.0	2.7	*92.0*	4.9	0.4
Allowing infants to sleep in the same room as their parents	*31.0*	56.2	5.6	5.1	2.2
Placing infants for sleep in a prone position	5.1	0.7	*92.8*	0.5	0.9
Breastfeeding	*82.6*	14.9	0.0	2.2	0.4
Performing an electrocardiogram on the infant	9.1	*83.0*	0.0	6.9	1.1
Placing soft objects such as pillows, quilts and stuffed toys in the crib	0.0	5.1	*91.5*	2.9	0.5
Smoking (both maternal and paternal) in the infant’s environment	0.0	1.3	*96.9*	1.5	0.4
Sleeping with an infant on a couch/armchair	0.7	18.3	*66.7*	13.2	1.1

Percentages may not add up to 100% because of rounding

In Table 6 we can see the relationship between the variables of interest and the significant explanatory variables. The respondents’ knowledge about SIDS risk factors (expressed as the proportion of correct answers over the 13 items included in the survey) was positively correlated with how the respondents rated their own knowledge on this topic and their confidence in discussing SIDS related issues with parents (ρ = 0.233 and ρ = 0.207, p < 0.001 in both cases). Paediatricians’ perception of being qualified to advise parents and make recommendations about SIDS was also positively correlated with their knowledge (ρ = 0.160, p < 0.001), while there was a negative correlation with seniority (ρ = −0.157, p < 0.001). The respondents’ ratings of their own knowledge and their confidence in discussing issues related to SIDS were also positively correlated with the other variables of interest: being aware that the supine position is the safest sleep position and exclusively recommending the supine position to parents. In the first case correlations were of 0.124 and 0.126 (p < 0.01 in both cases), while in the second they were 0.123 and 0.174 respectively (p < 0.01 in both cases). Additionally, in the case of a correct knowledge about the safest sleep position we observed a negative correlation with having received a specific training about SIDS (ρ = −0.166, p = 0.021), while in the case of correct recommendations about the safest sleep position we could see a negative correlation for those respondents working in a private clinic (ρ = −0.191, p = 0.021).

**Table 6:** Correlation between selected covariates and the variables of interest: respondents’ knowledge about SIDS risk factors (expressed as proportion of correct answers over the 13 items which were considered), respondents’ knowledge about the safest sleep position (correct or not), and respondents’ recommendations to parents about the safest sleep position (correct or not)

	Knowledge about SIDS risk factors (proportion of correct answers over 13 items)		Correct knowledge about the safest sleep position		Correct recommendations about the safest sleep position	
	Correlation coefficient (ρ)^a^	Significance	Correlation coefficient (ρ)^a^	Significance	Correlation coefficient (ρ)^a^	Significance
Seniority (years of practice, continuous)	−0.157	< **0.001**	−0.007	0.880	−0.031	0.479
Years since latest training about SIDS	−0.087	0.237	−0.052	0.485	−0.027	0.720
Confidence in discussing issues related to SIDS with parents (from 1 = very low to 5 = very high)	0.207	<**0.001**	0.126	**0.003**	0.174	<**0.001**
Self-assessed knowledge about SIDS and its risk factors (from 1 = very low to 5 = very high)	0.233	<**0.001**	0.124	**0.004**	0.123	**0.005**
Has received specific training about SIDS	−0.024	0.578	−0.166	**0.021**	−0.078	0.308
Workplace: CAP	0.013	0.764	0.015	0.859	−0.064	0.372
Workplace: Private clinic	−0.015	0.724	−0.114	0.182	−0.191	**0.021**
Workplace: Private practice	0.016	0.705	−0.001	0.999	−0.077	0.296
Workplace: Public hospital	−0.038	0.370	0.029	0.699	0.128	0.097
Workplace: Private hospital	0.020	0.637	−0.096	0.325	−0.103	0.262
Perceives to be qualified to advise parents and make recommendations about SIDS	0.160	<**0.001**	0.096	0.420	0.129	0.287
Has direct experience of a case of SIDS	0.048	0.265	0.028	0.717	0.062	0.410
Has children	0.013	0.757	−0.085	0.333	−0.091	0.328
Has children aged 3 or less	0.056	0.192	0.019	0.881	0.134	0.227

Bold indicates significant values which are < 0.05, and are thus statistically significant at 95%

^a^Pearson’s correlation was performed when both variables were continuous; Spearman’s correlation was performed when one variable was ordinal and the other was either continuous or dichotomous; point biserial correlation was performed when one variable was continuous and the other was dichotomous; tetrachoric correlation was performed when both variables were dichotomous

## Discussion

The response rate to this survey (45.9%) was not far from the 54% which Asch and colleagues reported as the average response rate for mail surveys of physicians (Asch et al. 1997), and placed this study well above the average response rate registered by similar surveys on this topic (27.7%, de Luca and Hinde 2016). However, even if 63.8% of respondents had a direct experience of a case of SIDS, our results showed that paediatricians devoted little time to SIDS. Their training on this topic seemed insufficient, since only 34.4% attended a specific training course. On average, this training took place eight years before this survey and almost 40% of respondents were not satisfied with it. Considering the crucial role that the Strategic Plan for Paediatric Primary Care attributes to paediatricians in Catalonia in terms of transmitting the SIDS risk reduction message to parents (Generalitat de Catalunya 2007), respondents reported discussing SIDS with parents less often than it was reasonable to expect: in more than 50% of the cases, in fact, they reported doing so less than once a week, although this percentage decreased to about 20% if we focused on how often they gave recommendations about the safest sleep position to parents. This remarkable difference between the two distributions suggests that respondents could be differentiating between conversations about SIDS and conversations about sleep position, thus not realizing that a conversation about the safest sleep position is, even if indirectly, a conversation about SIDS.

Paediatricians rated their own knowledge about SIDS as very high or somewhat high in 63.9% of the cases, and on average answered correctly to 75% of the risk factors items of the survey. However, only 57.6% of respondents recognized the supine position as the safest position against SIDS, and only 58.1% exclusively recommended the supine position to parents. These percentages are in line with those measured in Italy between 2008 and 2009 (de Luca and Vida 2014). In both studies, a significant proportion of respondents stated that the lateral position is also acceptable, which is surprising as we are considering a highly qualified population. Similar levels (64%) were also observed in the United States, but this was before the bulk of the prevention campaigns were carried out (Scheidt et al. 1993), while in more recent years the level reached about 82% (Moon et al. 2007b). The most immediate consequence of this situation was that a significant proportion of children did not get the most protective advice, which is highly undesirable. We observed that these percentages were not affected by standard demographic and professional variables (e.g., gender or seniority) or by those which could be proxies for a personal interest on the topic (e.g., having had a direct experience of a case of SIDS or having their own children). However, a significant positive correlation existed between paediatricians’ self-evaluation and their actual knowledge about this topic: the higher they rated themselves in terms of confidence or knowledge, the greater their actual knowledge about this topic was. Specifically, those who did not consider themselves as qualified to advise parents but still gave recommendations to them, had a level of knowledge which was on average 7 percentage points lower than the one of other paediatricians (76.1 against 69.1%, p = 0.009, data not shown in table). If properly encouraged under the right circumstances, this self- evaluation could lead those who do not feel confident or knowledgeable enough on this topic to seek specific training on SIDS in order to fill any gap they might have identified. This would also imply that paediatricians themselves might already have all the tools which would be needed to improve this situation.

When looking at the single risk factors presented in Table 5, we could see that 89.0% of paediatricians recognized that the supine position lowered the risk of SIDS and that 92.8% recognized that the prone position increased the risk of SIDS. However, non-negligible amounts of respondents (6.9 and 5.1%, respectively) stated that the supine position increased the risk and that the prone one contributed to lowering it, highlighting the need of further training also on items that nowadays may be considered as universally accepted. Other key items related to the sleeping conditions such as bed or sofa sharing registered about 70% of correct answers. Specifically, 66.7% of respondents knew that sofa sharing represented a risk factor for SIDS, while 18.3% stated that it did not have any effect on the risk of SIDS. An additional 13.2% recognized that they did not know the answer to this item (the highest level of uncertainty when evaluating a proven risk factor), which may suggest that this behaviour did not receive enough attention within the context of the SIDS risk reduction message. 73.2% of paediatricians recognised bed sharing as a risk factor for SIDS, while 16.5% believed that this behaviour had no impact on the risk of SIDS. It needs to be noted though, that in recent years the exact role of bed sharing (or co-sleeping) has been extensively debated. While a few studies published after the AAP guidelines still concluded that bed sharing represented a risk factor for SIDS (Carpenter et al. 2012; Vennemann et al. 2012), the most recent ones suggested that it might not be a risk factor for SIDS per se. Instead, there would be specific hazardous bed sharing circumstances that significantly increase the risk of SIDS (e.g., if parents consume alcohol, smoke or take drugs, Fleming et al. 2015; Blair et al. 2014). The survey presented in this article was carried out before the studies of 2014 and 2015, and thus relied on the definition of bed sharing as a risk factor. However, for this specific item it should be kept in mind that some respondents might not necessarily have given the wrong answer out of ignorance but rather because they were aware of the issues surrounding this topic.

This survey also showed that having received a specific training about SIDS did not improve paediatricians’ knowledge about SIDS risk factors or the safest sleep position. Actually, it was detrimental for the knowledge about the safest sleep position. This might seem counter-intuitive, but could be explained by the fact that, on average, paediatricians attended the training 8 years before this survey, when the supine position was still not universally recognized as the unique in reducing the risk of SIDS in Spain. Seniority also had a moderate negative influence on the degree of knowledge about SIDS risk factors. Similarly to the previous consideration, it needs to be taken into account that most of SIDS risk factors were discovered (or updated and changed) quite recently (e.g., bed sharing, or even the recommended sleeping position itself). This circumstance might explain why younger paediatricians had a higher level of knowledge on this topic. In such a fast-moving field, trainings should be updated and taken regularly, so as to ensure that healthcare professionals deliver the latest evidence-based messages. If trainings are not updated regularly, there is a danger that they might lull professionals into a false sense of security, making them feel that they do not need to keep up with the latest developments on the grounds that they attended a training course, and so have been made aware of the best practice.

It should be pointed out that this study also has some limitations. The response rate was only 46%, which exposes it to a potential selection bias. In an effort to asses this risk, we observed some significant unbalance in terms of gender and age, but none of these variables played a role in determining paediatricians’ knowledge and recommendations on this topic. Some healthcare professionals may not necessarily have given the wrong answer out of ignorance but rather because they were aware of the latest issues surrounding some risk factors which are still debated by the scientific community (e.g., the different conditions of bed sharing). Due to space constraints, respondents were not given specific instructions about the definition of a ‘*direct experience of a case of SIDS*’, which may have led to different interpretations of the question by different respondents. In this context, future studies should also consider whether it might be appropriate to look at the more inclusive category of SUDI rather than only on SIDS, thus focusing on the knowledge about all sleep-related deaths targeted by the safe sleep recommendations. Moreover, the survey was affected by two typographical errors in the Catalan version. Another limitation is that the participation of the provinces in the project was not homogeneous, and it is important to remember that the study is centred on a single Spanish region. As a consequence, any extension of the conclusions to a national level would not be justified.

## Conclusion

Overall, efforts should be made in order to improve paediatricians’ knowledge and practice about the SIDS risk reduction message. In the light of our results, specific refresher trainings about SIDS and its risk factors are highly recommended, and should especially target paediatricians with higher seniority. These trainings could be provided as optional modules, as paediatricians seemed to be fully aware of their degree of knowledge (high or low) and could as well recognize an eventual need of an update. Active public health policies in reducing the risk of SIDS, endorsed by scientific societies, would contribute to the dissemination of knowledge about SIDS and help lowering its rate.
